# Identification of tRNA nucleoside modification genes critical for stress response and development in rice and *Arabidopsis*

**DOI:** 10.1186/s12870-017-1206-0

**Published:** 2017-12-21

**Authors:** Youmei Wang, Chaoqun Pang, Xukai Li, Zhen Hu, Zhengyi Lv, Bo Zheng, Peng Chen

**Affiliations:** 1 Biomass and Bioenergy Research Centre, Huazhong Agricultural University, Wuhan, 430070 China; 2 College of Plant Science and Technology, Huazhong Agricultural University, Wuhan, 430070 China; 3 Key Laboratory of Horticultural Plant Biology of Ministry of Education, Huazhong Agricultural University, Wuhan, 430070 China; 4 College of Horticulture and Forestry Sciences, Huazhong Agricultural University, Wuhan, 430070 China; 5 College of Life Sciences, Shanxi Agricultural University, Taigu, Shanxi Province 030801 China

**Keywords:** tRNA, Modified nucleoside, Methyltransferase, Stress, Development

## Abstract

**Background:**

Modification of nucleosides on transfer RNA (tRNA) is important either for correct mRNA decoding process or for tRNA structural stabilization. Nucleoside methylations catalyzed by MTase (methyltransferase) are the most common type among all tRNA nucleoside modifications. Although tRNA modified nucleosides and modification enzymes have been extensively studied in prokaryotic systems, similar research remains preliminary in higher plants, especially in crop species, such as rice (*Oryza sativa*). Rice is a monocot model plant as well as an important cereal crop, and stress tolerance and yield are of great importance for rice breeding.

**Results:**

In this study, we investigated how the composition and abundance of tRNA modified nucleosides could change in response to drought, salt and cold stress, as well as in different tissues during the whole growth season in two model plants–*O. sativa* and *Arabidopsis thaliana*. Twenty two and 20 *MTase* candidate genes were identified in rice and *Arabidopsis*, respectively, by protein sequence homology and conserved domain analysis. Four methylated nucleosides, Am, Cm, m^1^A and m^7^G, were found to be very important in stress response both in rice and *Arabidopsis*. Additionally, three nucleosides,Gm, m^5^U and m^5^C, were involved in plant development. Hierarchical clustering analysis revealed consistency on Am, Cm, m^1^A and m^7^G *MTase* candidate genes, and the abundance of the corresponding nucleoside under stress conditions. The same is true for Gm, m^5^U and m^5^C modifications and corresponding methylation genes in different tissues during different developmental stages.

**Conclusions:**

We identified candidate genes for various tRNA modified nucleosides in rice and *Arabidopsis*, especially on *MTases* for methylated nucleosides. Based on bioinformatics analysis, nucleoside abundance assessments and gene expression profiling, we propose four methylated nucleosides (Am, Cm, m^1^A and m^7^G) that are critical for stress response in rice and *Arabidopsis*, and three methylated nucleosides (Gm, m^5^U and m^5^C) that might be important during development.

**Electronic supplementary material:**

The online version of this article (10.1186/s12870-017-1206-0) contains supplementary material, which is available to authorized users.

## Background

As indispensable participants in protein synthesis, tRNAs are highly modified in both prokaryotes and eukaryotes. The genes that encode tRNA modification enzymes constitute far more than the tRNA coding genes, which further highlights the importance of tRNA modification [1, 2]. Besides the canonical functions in protein synthesis, emerging evidence emphasized critical role for tRNA nucleoside modification in regulation of cellular response to stimuli and developmental signals [2–7]. Previous studies in bacteria, yeast and animal systems illustrated various tRNA modified nucleosides affecting bacterial virulence, yeast exocytosis, *C.elegans* embryo development and human mitochondrial diseases such as MELAS and MERRF [7–13].

However, we understand quite poorly about the function of tRNA nucleoside modifications on either development or stress response in higher plants. Plants are particularly prone to environmental changes such as drought, cold and high salinity stresses. Research of tRNA nucleoside modification changes upon various stresses or during development is scarce in higher plants. In *Arabidopsis*, a few components of the Elongator complex (*ELP1–6*, *KTI11–14*) were shown to regulate cell proliferation, anthocyanin biosynthesis, drought stress tolerance and immune response [14–18]. These genes participated in ncm^5^U (5-carbamoylmethyluridine) modification at position 34 on certain tRNA species [19–21]. In addition to the *elp* mutants, certain *Arabidopsis* mutants of tRNA nucleoside modification genes also showed mild phenotypes, such as slow growth of *smo2* [22] and reduced respiration rate and less fresh weight of seedlings in *tad1* [23]. It has been long postulated that modified nucleosides on tRNA molecules may function as “biosensor” for environmental and physiological changes [24, 25], as a fast module to regulate gene expression at translational level. In agreement with this hypothesis, the abundance of tRNA modified nucleosides do change in response to various stresses [26]. The mutants of *AtTRM4B*, m^5^C (5-methylcytidine) methylation modification gene, had shorter primary roots and increased sensitivity to oxidative stress [27]. Our recent study showed that Am nucleoside (2’-O-methyladenosine) was induced by salt stress in a variety of plants including Rice, Poplar, *Arabidopsis* and Brachypodium [28]. Overexpression of *OsTRM13*, the corresponding gene for Am and Cm modification at position 4, improved salt stress tolerance in rice [28].

Among the hundreds of modified nucleosides present on tRNA molecules, methylation is the most ubiquitous and abundant type [29–31]. Nucleoside methylation are catalyzed by methyltransferases (MTases) [30, 31]. tRNA MTases in *S. cerevisiae* are mostly AdoMet-dependent methyltransferase, either in RFM superfamily or SPOUT superfamily [32–36]. Another method of classification of AdoMet-dependent MTases was suggested by *Schubert HL* et al. by using the catalytic domain as criteria for function annotation [37]. MTases for tRNA nucleoside methylations in *S. cerevisiae* were considered as good references for the study of tRNA MTases in higher plants. Although a few tRNA MTase in *Arabidopsis* have been reported [22, 27, 38], little is known in cereal crops such as rice. The PlantRNA database (http://plantrna.ibmp.cnrs.fr/plantrna/) provides decent amount of information on tRNA genomic sequences and processing enzymes (such as the 5′ /3′ processing and intron-splicing enzymes), but not information for the exact location of modified nucleosides in tRNA sequences, either in *Arabidopsis* or in rice. As for the function of tRNA nucleoside modification enzymes, very few were supported with experimental data in *Arabidopsis* [22, 23, 38, 39], and none in *O. sativa*.

The detection and quantification of tRNA modified nucleosides provide a basis for studying the function of modifying enzymes. In comparison with the HPLC method used before for nucleoside quantification, Liquid chromatography-coupled tandem quadrupole mass spectrometry (LC-MS/MS) is a powerful tool with higher sensitivity for modified nucleosides quantification [6, 40]. In this study, LC-MS/MS was used to quantify changes of tRNA modified nucleosides under stress conditions as well as in different tissues along plant development in rice and *Arabidopsis*. 25 known tRNA nucleosides can be clearly separated and detected (Additional file 5: Figure S1), among which 12 were methylated nucleosides catalyzed by different TRM enzymes as MTases or associated partners (Additional file 5: Figure S2). With protein sequence homology, we identified candidate genes for a subset of methylated nucleosides. Hierarchical clustering was performed on abundance of modified nucleoside and transcript level of modification candidate genes from developmental or stress dataset. The results indicated that, besides Am nucleoside, other methylated nucleosides such as Cm, m^1^A (1-methyladenosine), and m^7^G (7-methylguanosine) might participate in regulation of stress response; whereas Gm (2’-O-methylguanosine), m^5^U (5-methyluridine) and m^5^C (5-methylcytidine) most likely participated in regulation of pant development.

## Results

### Identification of tRNA MTase candidate genes in rice and *Arabidopsis*

*O. sativa* and *A. thaliana* were selected for the monocot or the dicot model organism. tRNA MTase candidate genes were identified based on protein sequence homology with yeast tRNA MTases. 13 Trm proteins involved in methylated nucleosides in *S. cerevisiae* were used as query sequences, for blastp search of the rice and *Arabidopsis* candidates (Table 1, Additional file 4). Figure S2 (Additional file 5: Figure S2) showed the name of the yeast *TRM* genes, name and structure of the corresponding nucleosides, and the location of these methylated nucleosides on tRNA molecules. Trm1 for N2,N2-dimethylguanosine (m^2^_2_G) modification; Trm9 for the last step of 5-methyloxy carbonyl-methyluridine (mcm^5^U) and 5-methyl-amino-methyluridine (mnm^5^U); Trm3 and Trm7 for 2’-O-methylguanosine (Gm) and 2’-O-methylcytosine (Cm) respectively; Trm13 for 2’-O-methyladenosine (Am); Trm2 for 5-methyluridine (m^5^U), Trm11 and Trm112 form a complex for 2-methylguanosine (m^2^G); Trm5 and Trm10 for 1-methylguanosine (m^1^G) at position 37 and position 9, respectively; Trm8 and Trm82 from a complex for 7-methylguanosine (m^7^G); Trm6 and Trm61 for 1-methyladenosine (m^1^A); Trm4 for 5-methylcytidine (m^5^C), and Trm140/ABP140 for 3-methylcytidine (m^3^C) modification at position 32 of yeast tRNA [41] We did not quantify m^3^C in this study, since we only got one unique peak (Additional file 5: Figure S1) under *258/126 m/s (Q1/Q3)* which overlapped with the m^5^C nucleoside standard. Trm6-Trm61 is a two-subunit complex responsible for m^1^A58 in tRNA_i_^Met^, tRNA^Asn^_GUU,_ tRNA^Arg^_ACU_ and a few other isoacceptors [42, 43]. However, TRM61 is a direct MTase with conserved AdoMet-binding domain. This binding domain was not found in TRM6 protein, indicating TRM6 is accessory protein that may play a role in maintaining the stability of the complex [44, 45]. Similarly, TRM82 is a noncatalytic subunit of the heterodimeric complex TRM8-TRM82, which catalyzes m^7^G formation at position 46 [46]. TRM112 is a 15-KDa zinc-finger protein that assisted TRM9 and TRM11 for mcm^5^U and m^2^G methylation, respectively [47, 48].

**Table 1:** *Arabidopsis* and rice candidate genes for tRNA nucleoside methylation

Yeast Protein	Modification	*Arabidopsis* Candidate Gene	E-value	Rice Candidate Gene*	E-value	Note
Trm1	m^2^_2_G	At3g02320 (AtTRM1a)	1.0E-74	LOC_Os03g57280 (OsTRM1a)	1.1E-62	
		At5g15810 (AtTRM1b)	2.0E-73	LOC_Os10g21360 (OsTRM1b)	3.8E-62	
		At3g56330 (AtTRM1c)	2.0E-21	LOC_Os05g25870 (OsTRM1c)	3.1E-07	
Trm2	m^5^U	At3g21300 (AtTRM2a)	1.0E-23	LOC_Os01g09750 (OsTRM2a)	9.9E-24	
		At2g28450 (AtTRM2b)	9.0E-09	LOC_Os04g01480 (OsTRM2b)	3.5E-10	
Trm3	Gm	At4g17610 (AtTRM3)	7.0E-35	LOC_Os03g01110 (OsTRM3)	9.3E-29	
Trm4	m^5^C	At4g40000 (AtTRM4a)	e-101	LOC_Os09g29630 (OsTRM4a)	1.2E-100	
		At2g22400 (AtTRM4b)	2.0E-97	LOC_Os08g37780 (OsTRM4b)	9.8E-95	
		At5g55920 (AtTRM4c)	1.0E-21	LOC_Os02g49270 (OsTRM4c)	6.0E-24	
		At4g26600 (AtTRM4d)	2.0E-19	LOC_Os09g37860 (OsTRM4d)	4.7E-23	
		At3g13180 (AtTRM4e)	4.0E-14	LOC_Os08g27824 (OsTRM4e)	1.1E-10	
		At1g06560 (AtTRM4f)	9.0E-10	LOC_Os02g21510 (OsTRM4f)	4.5E-10	
		At5g66180 (AtTRM4g)	3.0E-08	LOC_Os02g12600 (OsTRM4g)	1.5E-06	
		At5g26180 (AtTRM4h)	2.0E-08			
Trm5	m^1^G	At3g56120 (AtTRM5a)	3.0E-58	LOC_Os01g29409 (OsTRM5a)	3.7E-60	
		At4g27340 (AtTRM5b)	4.0E-53	LOC_Os02g39370 (OsTRM5b)	2.4E-41	
		At4g04670 (AtTRM5c)	4.0E-06			
Trm6	m^1^A	At2g45730 (AtTRM6)	4.0E-03	LOC_Os04g02150 (OsTRM6)	8.3E-18	not MTase
Trm7	Cm	At5g01230 (AtTRM7a)	4.0E-79	LOC_Os06g49140 (OsTRM7a)	1.5E-74	
		At4g25730 (AtTRM7b)	1.0E-28	LOC_Os05g49230 (OsTRM7b)	2.3E-34	
		At5g13830 (AtTRM7c)	8.0E-20	LOC_Os09g27270 (OsTRM7c)	7.4E-17	
				LOC_Os03g60750 (OsTRM7d)	2.8E-15	
Trm8	m^7^G	At5g24840 (AtTRM8a)	8.0E-76	LOC_Os06g12990 (OsTRM8a)	4.1E-65	
		At5g17660 (AtTRM8b)	6.0E-08	LOC_Os01g35170 (OsTRM8b)	3.1E-06	
Trm9	mcm5 U	At1g36310 (AtTRM9)	2.0E-34	LOC_Os02g51490 (OsTRM9)	9.3E-42	
Trm10	m^1^G	At5g47680 (AtTRM10)	1.0E-29	LOC_Os02g49360 (OsTRM10)	1.5E-28	
Trm11	m^2^G	At3g26410 (AtTRM11)	2.0E-53	LOC_Os02g35060 (OsTRM11)	2.0E-54	
Trm13	Am	At4g01880 (AtTRM13)	5.0E-24	LOC_Os03g61750 (OsTRM13)	1.6E-09	
Trm61	m^1^A	At5g14600 (AtTRM61)	7.0E-47	LOC_Os04g25360 (OsTRM61a)	2.8E-44	
				LOC_Os04g25990 (OsTRM61b)	1.5E-43	
				LOC_Os05g07830 (OsTRM61c)	1.2E-15	
Trm82	m^7^G	At1g03110 (AtTRM82)	6.0E-11	LOC_Os03g53530 (OsTRM82)	2.2E-09	not MTase
Trm112	m^2^G	At1g78190 (AtTRM112a)	2.0E-11	LOC_Os07g43020 (OsTRM112)	2.0E-12	not MTase
		At1g22270 (AtTRM112b)	3.0E-07			
Trm140	m^3^C	At2g26200 (AtTRM140a)	1E-48	LOC_Os03g04940 (OsTRM140a)	3.4E-35	
		At1g54650 (AtTRM140b)	2E-28	LOC_Os07g23169 (OsTRM140b)	1.8E-25	

As shown in Table 1, 33 candidate genes were identified in both *O. sativa* and *A. thaliana,* respectively, with a blastp cutoff value at 1.0E-06 (Table 1). A further analysis by PFAM database confirmed the presence of conserved domain of the *TRM* genes, in both rice and *Arabidopsis* candidate genes (Table 2). Twenty two candidate genes were selected from rice and 20 from *Arabidopsis*, after a manual screen based on the domain structure similarity between the query sequences and the candidate genes (Table 2).

**Table 2:** Pfam analysis of *MTase* candidate genes in rice and *Arabidopsis*

Gene	Source	Accession	Description	Position	Gene	Source	Accession	Description	Position
(m^2^_2_G)Trm1	Pfam	PF02005	m22G tRNA meth_tr	33–490	(mcm^5^U)Trm9	Pfam	PF08241	Methyltransferase domain	50–141
AT3G02320	pfam	PF02005	m22G tRNA meth_tr	11–466	AT1G36310	pfam	PF08241	Methyltransferase type 11	112–202
AT5G15810	pfam	PF02005	m22G tRNA meth_tr	109–564	LOC_Os02g51490	HMMPfam	PF08241.5	Methyltransf_11	106–196
LOC_Os10g21360	pfam	PF02005.9	TRM	58–438					
LOC_Os03g57280	pfam	PF02005.9	TRM	19–476	(m^1^G)Trm10	Pfam	PF01746	tRNA (Guanine-1)-meth_tr	104–276
					AT5G47680	pfam	PF01746	tRNA (guanine-N1-)-meth_tr	128–293
(m^5^U)Trm2	Pfam	PF01938	(Uracil-5)-meth_tr	164–227	LOC_Os02g49360		PF01746.14	tRNA_m1G_MT	128–293
AT3G21300	pfam	PF05958	(Uracil-5)-meth_tr	354–552					
LOC_Os01g09750	HMMPfam	PF05958.4	tRNA_U5-meth_tr	366–554	(m^2^G)Trm11	Pfam	PF01170	Putative RNA methylase family UPF0020	184–297
					AT3G26410	pfam	PF01170	Putative RNA methylase	193–315
(Gm)Trm3	Pfam	PF00588	tRNA/rRNA meth_tr, SpoU	1286–1428	LOC_Os02g35060	pfam	PF01170.11	UPF0020	193–315
AT4G17610	pfam	PF00588	tRNA/rRNA meth_tr, SpoU	1700–1842					
LOC_Os03g01110	HMMPfam	PF00588.12	SpoU_methylase	1575–1716	(Am)Trm13	Pfam	PF11722	CCCH zinc finger in TRM13 protein	18–47
						Pfam	PF05253	U11-48 K-like CHHC zinc finger	71–97
(m^5^C)Trm4	Pfam	PF01189	NOL1/NOP2/sun family	254–581		Pfam	PF05206	Methyltransferase TRM13	179–473
At4g40000	pfam	PF01189	NOL1/NOP2/sun family	155–350	AT4G01880	pfam	PF05206	Methyltransferase TRM13	167–445
At2g22400	pfam	PF01189	NOL1/NOP2/sun family	163–361		pfam	PF05253	U11-48 K-like CHHC zinc finger domain	42–66
LOC_Os09g29630	HMMPfam	PF01189.10	Nol1_Nop2_Fmu	168–364		pfam	PF11722	Zinc finger, CCCH-type, TRM13	7–36
LOC_Os08g37780	HMMPfam	PF01189.10	Nol1_Nop2_Fmu	185–388	LOC_Os03g61750	pfam	PF11722.1	zf-TRM13_CCCH	20–49
						pfam	PF05253.5	zf-U11-48 K	61–86
(m^1^G)Trm5	Pfam	PF02475	tRNA transferase Trm5/Tyw2	177–436		pfam	PF05206.7	TRM13	181–247
AT3G56120	pfam	PF02475	tRNA transferase Trm5/Tyw2	116–411					
AT4G27340	pfam	PF02475	tRNA transferase Trm5/Tyw2	343–542	(m^1^A)Trm61	Pfam	PF08704	tRNA meth_tr complex GCD14 subunit	72–362
LOC_Os01g29409	HMMPfam	PF02475.9	Met_10	309–505	AT5G14600	pfam	PF08704	tRNA meth_tr complex GCD14 subunit	9–313
LOC_Os02g39370	HMMPfam	PF02475.9	Met_10	156–442	LOC_Os04g25360	HMMPfam	PF08704.3	GCD14	14–321
					LOC_Os04g25990	pfam	PF08704.3	GCD14	14–320
(Cm)Trm7	Pfam	PF01728	FtsJ-like meth_tr	21–207					
AT5G01230	pfam	PF01728	Ribosomal RNA meth_tr RrmJ/FtsJ	21–209	(m^3^C)Trm140	Pfam	PF08242	Methyltransferase domain	439–543
AT4G25730	pfam	PF01728	Ribosomal RNA meth_tr RrmJ/FtsJ	22–200	AT2G26200	pfam	PF08242	Methyltransferase type 12	79–180
AT5G13830	pfam	PF01728	Ribosomal RNA meth_tr RrmJ/FtsJ	19–223	AT1G54650	pfam	PF08242	Methyltransferase type 12	87–214
LOC_Os06g49140	HMMPfam	PF01728.12	FtsJ	21–209	LOC_Os03g04940	pfam	PF08242.5	Methyltransf_12	63–163
LOC_Os05g49230	HMMPfam	PF01728.12	FtsJ	22–201		pfam	PF10294.2	Methyltransf_16	326–485
LOC_Os09g27270	HMMPfam	PF01728.12	FtsJ	22–142	LOC_Os07g23169	pfam	PF08242.5	Methyltransf_12	96–229
LOC_Os03g60750	HMMPfam	PF01728.12	FtsJ	20–231					
(m^7^G)Trm8	Pfam	PF02390	Putative meth_tr	74–279					
AT5G24840	pfam	PF02390	tRNA (guanine-N-7) meth_tr	48–246					
AT5G17660	pfam	PF02390	tRNA (guanine-N-7) meth_tr	115–305					
LOC_Os06g12990	HMMPfam	PF02390.10	Methyltransf_4	58–254					
LOC_Os01g35170	HMMPfam	PF02390.10	Methyltransf_4	2–183					

### The tRNA nucleoside MTase candidate genes show an uneven distribution both in *Arabidopsis* and rice genomes

**Fig. 1:**
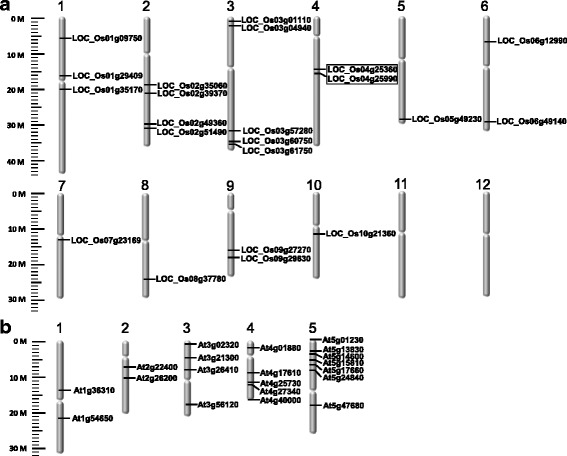
Physical map of tRNA nucleoside methyltransferase candidate genes on rice (a) or *Arabidopsis* (b) genomes. **a**, Chromosome location of 22 rice MTases candidate genes. **b**, Chromosome location of 20 *Arabidopsis* MTases candidate genes. Chromosome size are indicated by relative lengths. Tandemly duplicated genes are indicated by boxes

The chromosomal location of the rice or *Arabidopsis* tRNA nucleoside *MTase* candidate genes were shown in Fig. 1. Chromosome sizes and the position of each gene can be estimated by the scale on the left. The chromosomal distribution patterns showed that some chromosomes and chromosomal regions had a relatively high distribution of MTase genes. For example, the 22 rice *TRM* candidate genes were mapped to 10 out of 12 rice chromosomes and 54.5% of them were anchored on chr1, chr2 and chr3, whereas one only on chr.5, chr.7, chr.8 and chr.10, respectively, and no MTase gene was located on chr.11 or chr.12 (Fig. 1a). LOC_Os04g25360 and LOC_Os04g25990 located on chr.4 (marked out by the wireframe) were tandem duplicated gene pairs homologous to *Trm61* (Fig. 1a). In *Arabidopsis*, TRM candidate genes were found to be located more frequently on chr.4 and chr.5 (12 of 20), especially within a ca.10 Mb segment region on chr.5 (Fig. 1b). On the contrary, only two *TRM* candidates were mapped to chr.1 or chr.2 (Fig. 1b). No tandem duplicated gene pairs were found among *Arabidopsis MTase* candidate genes.

### Phylogenetic and conserved motif analysis of MTase in rice and *Arabidopsis*

**Fig. 2:**
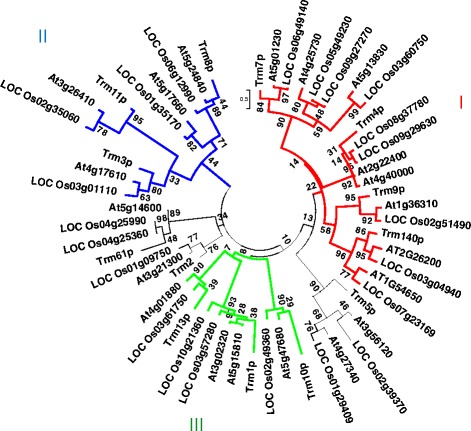
Circular neighbor-joining (N-J) tree of rice and *Arabidopsis* MTase candidate genes. Supporting values from bootstrap analysis were shown for each branch. The three groups of Trm proteins clustered together were annotated with red lines for group I, blue lines for group II and green lines for group III, respectively

Multiple sequence alignment was employed to construct a phylogenic tree of the tRNA nucleoside MTase candidates in rice and *Arabidopsis*, together with the 13 Trm query proteins in yeast (Fig. 2).

As shown in Fig. 2, MTases that modify analogous substrates were closely clustered: the three MTase groups related to cytidine modification (Trm4p, Trm140p for m^5^C and m^3^C respectively, and Trm7p for Cm modification) were clustered in group I; also found in group I was Trm9p which participated in the last step of mcm^5^U methylation. However, Sequence-structure-function analysis of tRNA m^5^C methyl-transferase Trm4p and RNA m^5^U methyltransferase revealed that RNA:m^5^C MTases shared a number of common features with the RNA:m^5^U MTases [49], suggesting a tentative evolution route of the two groups of 5-methylpyrimidine MTases. On the contrary, group II and group III MTases were responsible for methylpurine modifications: Trm3p, Trm8p and Trm11p corresponding to Gm, m^7^G and m^2^G in group II, and Trm5p, Trm10p and Trm1p corresponding to m^2^_2_G and m^1^G methylations in group III. In between group II and III we found Trm61p and Trm13p relatives for Am and m^1^A methylations (Fig. 2).

**Fig. 3:**
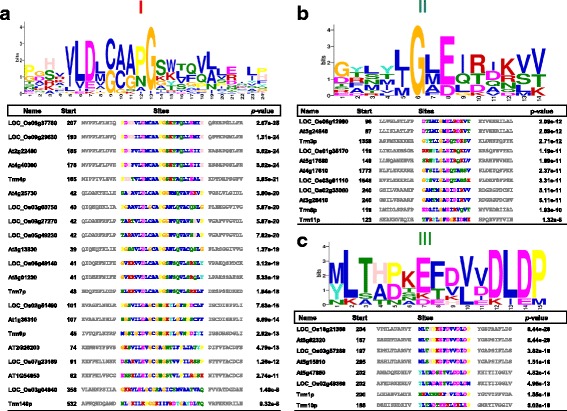
Conserved motif analysis of group I (**a**), group II (**b**) and group III (**c**) MTase candidate genes. X axis indicated position for each residue within the identified motifs, Y axis indicated bit score values. The size of the residue letter represented the degree of conservation within the group of proteins analyzed. The table below each graph illustrated the protein sequence for each member, with the name of the protein, starting position of the conserved motif and the whole motif sequence

Conserved motif analysis by MEME program [50] revealed the presence of conserved residues within the catalytic domain of group I, group II and group III MTases (Fig. 3). All four methyl-pyrimidine MTases (Trm4p, Trm7p, Trm9p, Trm140) exhibited a striking conservation of a Glycine (G) residue, as well as a consensus “VLD(L/M)CAA(P/N)” motif as its neighbor (Fig. 3a, Additional file 5: Figure S3). The strong conservation suggested that the Glycine residue and its surrounding motif most likely are involved in a structure that is required for substrate binding and/or catalysis. Similarly, a conserved pattern, the GxE/D motif was found in group II, from the Trm3p, Trm8p and Trm11p homologs (Fig. 3b). Previously, a crystal structure of the Trm8p revealed that E126 (as part of the conserved motif) is involved in a bidentate hydrogen bond with the ribose hydroxyl group [46]. Finally, a conserved DLD motif was found from Trm1p and Trm10p homologs from group III (Fig. 3c). In supporting of the functional relevance for this DLD motif, D211 from Trm1p of *S. cerevisiae* and D132 from Trmp1 of *Aquifex aeolicus* have been shown as the catalytic center for m^2^_2_G methylation [51, 52].

### Am, cm, m^1^A and m^7^G methylated nucleosides respond to stress treatment in rice and *Arabidopsis*

**Fig. 4:**
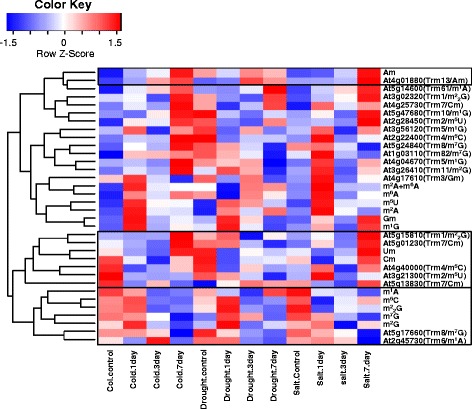
Hierarchical clustering analysis between tRNA nucleoside modification and the *Arabidopsis* candidate genes expression under stress conditions. Heat map was generated using raw data of nucleosides abundance or MTase candidate genes expression level under stress conditions, respectively, horizontally normalized and logarithmically transformed. LN2 value was presented in color key, which corresponded with ratio of change on either nucleoside level of the gene expression level under various stress conditions. A hierarchy-clustering was shown on the left, showing similar patterns between nucleosides and MTase candidate genes. Solid boxes indicated nucleosides and corresponding genes clustered together

**Fig. 5:**
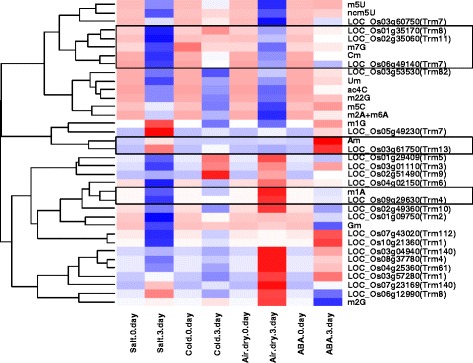
Hierarchical clustering analysis between tRNA nucleoside modification and the rice candidate genes expression under stress conditions. Heat map was generated using raw data of nucleosides abundance or MTase candidate genes expression level under stress conditions, respectively, horizontally normalized and logarithmically transformed. LN2 value was presented in color key, which corresponded with ratio of change on either nucleoside level of the gene expression level under various stress conditions. A hierarchy-clustering was shown on the left, showing similar patterns between nucleosides and MTase candidate genes. Solid boxes indicated nucleosides and corresponding genes clustered together

Hierarchical clustering analysis about tRNA nucleoside methylated modification and the candidate genes expression under stress tolerance was constructed (Fig. 4 and Fig. 5, Additional file 6). The amounts of methylated nucleosides under biotic and abiotic stress was quantified by LC-MS/MS method, in total 15 methylated nucleosides were quantified (marked in red color, Additional file 5: Figure S1). The gene expression profiles (Additional files 2 and 3) corresponding to tRNA methylation candidate genes were obtained from GEO DataSet (https://www.ncbi.nlm.nih.gov/gds/). Hierarchical clustering analyses revealed 4 methylated nucleosides (Cm, Am, m^1^A and m^7^G) with consistent changes on nucleoside level and the candidate methylation genes under stress tolerance (black wireframe, Fig. 4 and Fig. 5 Additional file 6). An obviously decrease of m^1^A and m^7^G nucleosides was observed under cold stress or salt stress, the level of Cm nucleoside was found to be decreasing under drought stress, whereas the level of Am nucleoside increased dramatically under salt stress in both rice and *Arabidopsis* (Fig. 4, Fig. 5, Fig. 7a, and b). In a recent study, we have shown that abscisic acid (ABA) treatment also induced a significant increase of Am nucleosides in rice seedlings [28].

### Three nucleosides gm, m^5^U and m^5^C are important in plant development

**Fig. 6:**
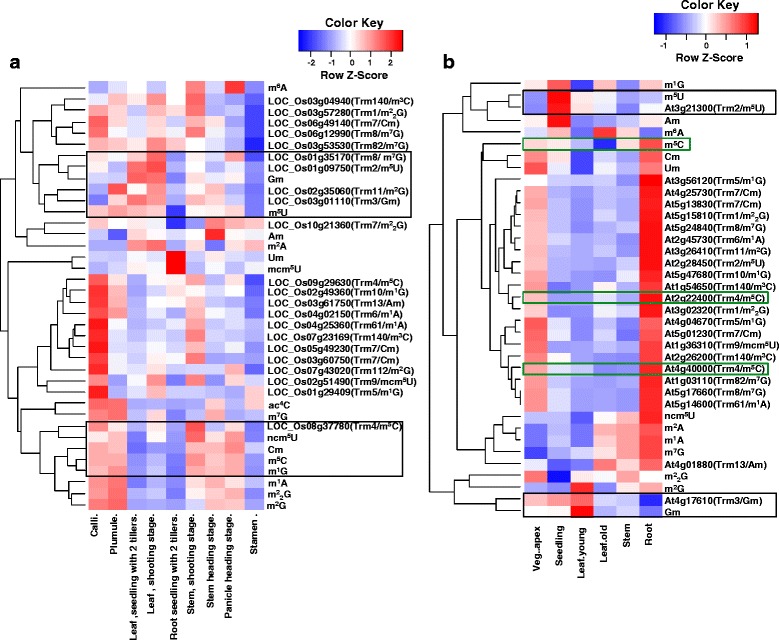
Hierarchical clustering analysis between tRNA nucleoside methylated modification and candidate genes expression level during development stage. Heatmap profiles about the abundance of methylated nucleosides and the expression level of the rice (**a**) or *Arabidopsis* (**b**) MTase candidate genes from developmental dataset. Heat map was generated from raw data of nucleoside levels and gene expression levels, normalized horizontally. LN2 values was shown above in color key. Solid boxes indicated nucleosides and the corresponding genes clustered into the same group. In panel B, green boxes indicated m^5^C nucleoside and the corresponding candidate genes in *Arabidopsis*. Solid boxes indicated nucleosides and corresponding genes clustered together

Nine different tissues in rice and six representative tissues of *Arabidopsis* were used to investigate tRNA nucleosides levels during the whole development period. Similar as in the stress dataset, the expression profile of methylated nucleoside candidate genes were obtained from the CREP database (http://crep.ncpgr.cn) for rice genes, and the TileViz database (http://jsp.Weigelworld.Org/tilevi-z/tileviz.jsp) for *Arabidopsis* genes (Additional file 1). A heat map was constructed based on the expression data and the levels of tRNA nucleosides in different tissues under various stages of development (Fig. 6, Additional file 6). Interestingly, three nucleosides Gm, m^5^U and m^5^C were clustered together with their corresponding candidate genes (Trm3p for Gm, Trm2p for m^5^U and Trm4p for m^5^C, respectively), both in rice (Fig. 6a) and in *Arabidopsis* (Fig. 6b). Many nucleoside modification genes in rice were found to be highly expressed in callus sample, and generally low levels in stamen tissues (Fig. 6A). In *Arabidopsis*, a high expression level of tRNA nucleoside modification candidate genes was found in root samples (Fig. 6b), a good consistency between m^5^C nucleoside and the corresponding candidate genes *AtTRM4a* (*At4g40000*) and *AtTRM4b* (*At2g22400*) were found in roots (Fig. 6b). The high level of m^5^C nucleoside and its corresponding methylation genes suggested a possible role in root development in *Arabidopsis*, which is consistent with recent findings of short root phenotype in *attrm4b* mutant [27].

**Fig. 7:**
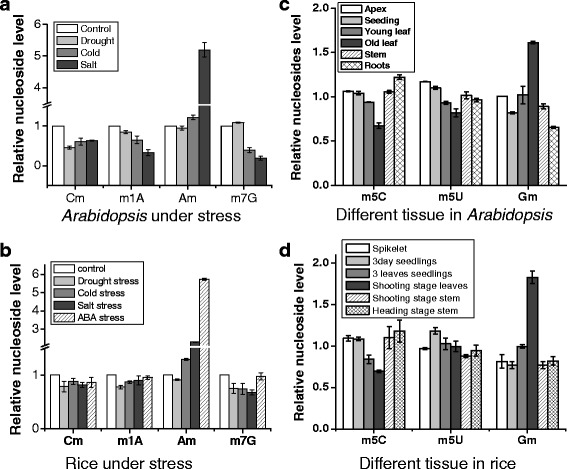
Selected methylated nucleosides that were potentially important for stress or development. (**a** and **b**) Quantification of Cm, m^1^A, Am, m^7^G nucleosides under stress conditions in *Arabidopsis* or rice. (**c** and **d**) Quantification of m^5^C, m^5^U and Gm nucleosides in different tissues during development

**Fig. 8:**
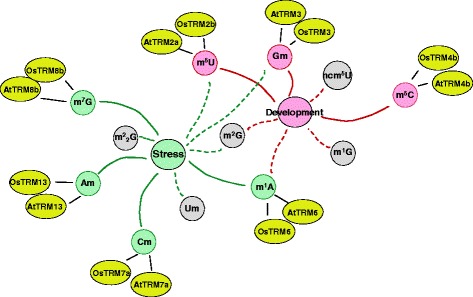
A model displaying the relationships within tRNA nucleoside methylation and stress/development. Green and red solid lines indicated nucleoside abundance and expression level of the corresponding MTase genes under stress or during development which could be clustered together, respectively. Green and red dashed lines showed nucleosides that changes under stress or during development, but shows no consistency with MTase gene expression

Figure 8 summarized methylated nucleosides associated with stress tolerance or development, with corresponding *MTase* candidate genes illustrated in yellow circles (Os_ for rice genes, At_ for *Arabidopsis* genes, Table 1). As mentioned above, Am, Cm, m^1^A and m^7^G nucleosides were regulated by stress (marked by green circle), in particular the Am nucleoside for salt stress and ABA treatment. The abundance of these nucleosides under stress conditions were clustered together (indicated by green lines in Fig. 8) with the expression level of the *MTase* candidate genes, i.e. *TRM13*, *TRM7*, *TRM61* and *TRM8* homologs in both rice and *Arabidopsis*. Another five nucleosides, m^5^U, Gm, m^2^G, Um and m^2^_2_G, also changed under stress conditions but showed no consistency with the expression level of the *MTase* candidate genes (gray circle, green dotted lines in Fig. 8). As for development, we found that three nucleosides Gm, m^5^C and m^5^U that might be important to plant development, these three nucleosides were clustered together with their *MTase* candidate genes (red circle, red solid lines in Fig. 8). m^5^C nucleoside abundance was high in young tissues, especially in roots. The abundance of Gm nucleoside increased in old leaves (Fig. 7c and d), indicating its potential role in leave senescence. Several other nucleosides such as m^2^G, m^1^A, m^1^G, ncm^5^U also showed tissue/organ specificity during development, but no consistency was found with the expression of their corresponding *MTase* candidate genes (gray circle, red dotted lines in Fig. 8).

## Discussion

In this study, we investigated the profile of tRNA modified nucleosides in different tissues and under various abiotic stress conditions in both rice and *Arabidopsis* seedlings. Rice is an important cereal crop and a monocot model plant. We identified 22 rice tRNA *MTase* candidate genes and 20 *Arabidopsis* genes by protein sequence homology to *S. cerevisiea*. Analysis of conserved motifs from the candidate MTases showed several types of conserved motifs including residues that might be involved in catalytic activity (Fig. 3). Also from motif analysis, three groups of MTases responsible for cytidine methylations, Trm4p (for m^5^C), Trm140(for m^3^C) and Trm7p (for Cm modification), were clustered together. A high conserved Glycine residue was identified from these groups of MTases (Fig. 3a), possibly indicating a role for Gly in structure required for substrate binding. In supporting of this, an isosteric mutation of K179 in Trm4p (neighbor to the conserved G177) was completely inactive [50]. G177 and K179 in Trm4p are located in motif I, which is regarded as AdoMet-binding site rather than the catalytic center. Likewise, G55 from Trm7p is also located in motif I predicted as AdoMet- and ribose-binding sites, while another conserved residue predicted by the motif analysis, D49 (Fig. 3a), is associated with binding to ribose and phosphate group of the nucleotide to be methylated [53]. The conserved G in Trm140p (G549) was also reported critical for AdoMet-binding. As for the second conserved D residue, point mutation of D547 in Trm140p resulted in decreased activity in m^3^C methylation [41]. Taken together, the high conserved domain in group I (Fig. 3a) most likely constitute for an AdoMet-binding site and indispensable for catalytic activity.

With protein sequence homology, we identified candidate genes for a subset of modified nucleosides from rice and *Arabidopsis* genomes. However, in half of the branches (Fig. 2) more than one candidate genes were present. This could be anticipated because gene duplication event is rather common in plant kingdom, additionally tissue-specific and subcellular organelles may demand for different set-up of tRNA nucleoside modification machinery. With hierarchical clustering analysis between gene expression and abundance of modified nucleosides in tissues and stress conditions, we found that not every candidate gene can cluster together with the modified nucleosides level. This could be explained in the following ways: 1) the unequal preference between redundant candidate genes, i.e. one of the redundant candidate genes might bear the primary modification activity whereas the other might be inactive. An example of this is the Trm4 homologs in *Arabidopsis*: *AtTRM4a* was shown to be inactive while *AtTRM4b* participated in the methylation at tRNA positions C48, C49, and C50 [27]. This is consistent with our result from hierarchical clustering analysis, that AtTRM4b(At2g22400) expression and the corresponding modified nucleosides m^5^C were clustered together under development (Fig. 6). 2) Insignificant or irregular changes in tRNA modified nucleosides and the expression of corresponding candidate genes. In other word, these nucleosides may hardly be regulated by drought, cold or salt stress.

From hierarchical clustering, we propose that certain methylated nucleosides, namely Am (2′-methyladenosine), Cm (2’-O-methylcytidine), m^1^A (1-methyladenosine), and m^7^G(7-methylguanosine) were involved in plant stress responses, while Gm(2’-O-methylguanosine), m^5^U (5-methyluridine), m^5^C (5-methylcytidine) in plant development. With the corresponding MTase genes identified in rice and *Arabidopsis*, it is possible to verify the function of these candidate genes in regulation of certain type of abiotic stress, or in general physiology for plant growth and development. Actually, our preliminary result of several *Arabidopsis* candidate genes suggested these candidate genes do have a role in stress response or root growth, our recent finding of OsTRM13 in salt stress tolerance in rice also suggests that the function of tRNA nucleoside modification and modification genes is yet to be illustrated.

## Conclusion

We showed in this study that both physiological conditions and environmental stimuli led to changes in tRNA modified nucleosides, these changes were accompanied by regulation on the expression of corresponding modification genes. By quantification of both the level of methylated nucleosides and the candidate MTase genes, under drought, cold and high salinity stresses in rice and *Arabidopsis*, we found four methylated nucleosides (Am, Cm, m^1^A, and m^7^G) critical for stress response and similarly three nucleosides (Gm, m^5^U and m^5^C) for plant development. All results showed good consistency in rice and *Arabidopsis*. This highlighted future work for transgenic manipulation and functional study of the corresponding modification enzymes. Phylogeny and motif analysis also suggested conserved residues within each group of MTases, those residues may be important for catalytic activity which awaits to be tested by future work.

## Methods

### Plant material and growth conditions

Nipponbare rice (*O. sativa* cv. Japonica) seeds were germinated in sterilized water for 2 days and then grown hydroponically in a climate chamber with a 16 h-light /8 h-dark cycle at 28 °C. At 3-leaves stage (2–3 weeks after germination), seedlings were transferred to the field located at HuaZhong Agricultural University, Wuhan, HuBei Province (latitude N31°, longitude E115°, average altitude 27 m). The growth season starts from April–May and ends around August–September.

*Arabidopsis* in *Colombian-0* background was used in this study. The sterilized seeds were sowed on 1/2 MS medium and grown in a culture room at 22 °C after vernalization, with 16 h light/8 h dark photo period at 4 °C. 10 days old seedlings were transplanted in soil, growing in a growth chamber with similar conditions.

### Sampling of tissues during the whole growth season

Mature rice seeds were sterilized with 70% ethanol followed by 2.5% sodium hydrochloride, washed and soaked in distilled water for 2 days. Plumule and radicle were sampled after seed germination in the dark for 48 h. Seedlings were grown hydroponically in the climate chamber with the settings abovementioned. The following tissue samples were taken during the cultivation in the climate chamber: 3d–seedling and 3d–root taken 3 days after germination, 3-leaf seedling and corresponding root taken 2 weeks after germination. Samples of leaf, stem and root during the shooting stage (about 70 days after sowing), heading stage (about 90 days after sowing) and flowering stage (85–95 days after sowing) were taken after transferring seedling to the field. In addition, panicle, spikelet and anther samples were also taken during flowering stage. A callus sample was taken from MS medium after induction and two rounds of sub-culturing. All samples were taken with three biological duplicates, flash frozen and stored in −80 °C for further use.

For *Arabidopsis* sampling, roots were sampled 9–10 days after seed germination. Other samples were taken through the whole development stage as following: apex (seed germination for 1-2 day), seedling (germination for 7 day), young leaf (4-leaves stage), old leaf (rosette leaves in flowering stage), stem (base stems in flowering stage). All samples were taken with three biological replicates, flash frozen and stored in −80 °C for further use.

### Stress treatment

For rice stress treatment, 9-d-old seedlings from climate chamber were used as starting materials. Cold stress was applied by putting rice seedlings into pre-equilibrated 4 °C cold water, samples were taken 0 d, 1 d, 3 d, 5 d, and 7 d after the treatment. A control set was taken with normal growth conditions. Drought (air dry) stress was applied by transferring seedlings onto 3MM filter paper under room temperature; samples were taken at the same time points as above. For high salinity stress, distilled water was changed into 200 mM NaCl for hydroponic cultivation and samples taken 0d, 1d, 3d, 5d, and 7d after salt stress. Samples were taken in triplicates, each sample with 15–20 seedlings.

Twenty one days old *Arabidopsis* Col.0 seedlings were used as starting materials for stress treatment. Drought stress was applied by water withholding and samples were taken at 0 d, 3 d, 7 d after the treatment. Cold stress and salt stress were applied in similar ways as for rice seedlings described above, a control set was taken with normal growth conditions. All samples were taken in triplicates, each sample with 5–10 seedlings.

### tRNA isolation and nucleoside analysis by *LC-MS/MS*

Small RNAs (< 200 bp) were extracted using microRNA Extraction Kit (Omega Bio-tek Inc.), digested into nucleosides and analyzed by LC-MS/MS with an LC-20A HPLC system and a diode array UV detector (190-400 nm). Details for LC-MS/MS analysis was described previously [28]. The abundance of each nucleoside under stress conditions was normalized to that in control set, the ratio (fold of change) of each nucleoside was calculated and used for heatmap generation.

### Database search for rice and *Arabidopsis* tRNA nucleoside modification candidate genes and bioinformatic analysis

The protein sequences of known tRNA nucleoside MTases from *S. cerevisiae* were used as query sequences, for retrieval of rice gene homologs with Blastp search tool on RGAP database (http://rice.plantbiology.msu.edu/index.shtml), or for *Arabidopsis* gene on TAIR database (http://www.arabidopsis.org/). A cut-off value was set as 1.0E-6 for initial identification of candidate genes. Protein sequences were manually verified by protein domain analysis on pfam (http://pfam.xfam.org/). The logos and conserved motifs were identified by MEME online searching engine (http://meme-suite.org/tools/meme) with parameters setup as the following: motif site distribution, any number of repetitions; maximum number of motifs, 5; motif width, from 8 to 50. Conserved motifs were verified on ESPript 3.0 (http://espript.ibcp.fr/ESPript/cgi-bin/ESPript.cgi) with an ALN file constructed by ClustalX 2.0.

All verified MTase genes were mapped to the *O. sativa* or *A. thaliana* genome chromosomes respectively with tool@Oryabase (http://viewer.shigen.info/ory-zavw/maptool/Map-Tool.do) for rice and Chromosome Map Tool (http://www.arabidopsis.org/jsp/ChromosomeMap/tool.jsp) for *Arabidopsis*, in addition to information from the RGAP and TAIR database. Non-rooted Neighborhood Joining tree of each family of MTases was conducted with MEGA4.0 software [54], bootstrap analysis was performed with 1000 iterations.

### Gene expression analysis of MTase candidate genes

Microarray data of MTase candidate genes under drought, salt, cold stress and ABA (ABA treatment only for rice) treatment were downloaded from GEO (Gene Expression Omnibus, http://www.ncbi.nlm.nih.gov/gds/), data series GSE6901, GSE37940, GSE26280 and GSE58603 for rice stress, and data series GSE72050, GSE80099, GSE53308, GSE16765, GSE71271 and GSE3326 for *Arabidopsis* stress. CREP database http://crep.ncpgr.cn provided the expression data for rice MTase genes of the whole development stage, the developmental datasets for *Arabidopsis* MTases were downloaded from TileViz (http://jsp.Weigelworld.Org/tilevi-z/tileviz.jsp).

Hierarchical clustering analysis was performed with gene expression and abundance of modified nucleosides from developmental or stress datasets. The heat map construction used heatmap.2, a software written by R language using Euclidean distance as the distance metric for agglomerative clustering. The absolute expression value from different tissues and under different stress conditions were first normalized by control experiment, the ratios (fold of change) of each candidate gene was calculated and used for heatmap generation.

## Additional files

Additional file 1: Expression data for *Arabidopsis* and rice MTase candidate genes of different development stage (XLSX 30 kb)

Additional file 2: Expression data for *Arabidopsis* MTase candidate genes under various stress conditions (XLSX 24126 kb)

Additional file 3: Expression data for rice MTase candidate genes under stress conditions (XLSX 35573 kb)

Additional file 4: Protein sequences of MTase candidate genes from *Arabidopsis* or rice (XLSX 27 kb)

Additional file 5: Figure S1. LC-MS/MS chromatogram in MRM mode for authentic nucleosides. The Y axis indicated signal intensity for a particular Q1 and Q3 ions. Numbered peaks are 1: U; 2: C; 3: G; 4: A; 5: D; 6: Ψ; 7:Cm; 8: m^5^C; 9: ac^4^C;,10: Am; 11: m^1^A; 12: m^2^A; 13: m^6^A; 14: m^6^t^6^A; 15: Gm; 16:m^7^G; 17: m^1^G; 18: m^2^G; 19:m^2^_2_G; 20: Um; 21: m^5^U; 22: ncm^5^U; 23: I; 24: m^1^I; 25: t^6^A. **Figure S2**. Eukaryotic tRNA and methylated nucleosides. (A) tRNA cloverleaf structure with methylated nucleosides shaded in gray. The abbreviated name of modified nucleosides was shown in Abbreviations section. (B) Chemical structure of methylated nucleosides and their corresponding MTase enzymes in *S. cerevisiae*. **Figure S3**. Multi sequence alignment of methyl-pyrimidine MTases in group I. The conserved amino acids were marked with blue boxes. A high conserved site Gly(G) was highlighted in red (PPTX 399 kb)

Additional file 6: Nucleoside abundance analysis for rice and *Arabidopsis* samples during stress or development (XLSX 30 kb)

## Abbreviations

ac^4^C: N4-acetylcytidine
Am: 2’-O-methyladenosine
Cm: 2’-O-methylcytidine
D: Dihydrouridine
Gm: 2’-O-methylguanosine
I: Inosine
m^1^A: 1-methyladenosine
m^1^G: 1-methylguanosine
m^1^I: 1-methylinosine
m^2^_2_G: N2, N2-dimethylguanosine
m^2^A: 2-methyladenosine
m^2^G: N2-methylguanosine
m^3^C: 3-methylcytidine
m^5^C: 5-methylcytidine
m^5^U: 5-methyluridine
m^6^A: N6-methyladenosine
m^6^t^6^A: N6-methyl-N6-threonylcarbamoyladenosine
m^7^G: 7-methylguanosine
mcm^5^U: 5-methoxycarbonylmethyluridine
ncm^5^U: 5-carbamoylmethyluridine
t^6^A: N6-threonylcarbamoyladenosine
Um: 2’-O-methyluridine
Ψ: Pseudouridine
