# Correction to: Identification of tRNA nucleoside modification genes critical for stress response and development in rice and *Arabidopsis*

**DOI:** 10.1186/s12870-018-1247-z

**Published:** 2018-02-19

**Authors:** Youmei Wang, Chaoqun Pang, Xukai Li, Zhen Hu, Zhengyi Lv, Bo Zheng, Peng Chen

**Affiliations:** 1 Biomass and Bioenergy Research Centre, Huazhong Agricultural University, Wuhan, 430070 China; 2 College of Plant Science and Technology, Huazhong Agricultural University, Wuhan, 430070 China; 3 Key Laboratory of Horticultural Plant Biology of Ministry of Education, Huazhong Agricultural University, Wuhan, 430070 China; 4 College of Horticulture and Forestry Sciences, Huazhong Agricultural University, Wuhan, 430070 China; 5 College of Life Sciences, Shanxi Agricultural University, Taigu, Shanxi Province 030801 China

## Correction

Following publication of the original article [1], the authors reported that there was a mistake in the presentation of their funding information. The sentence “This study was supported by the National Natural Science Foundation of China (31,100,268 to Peng Chen, 31,270,658 to Bo Zheng);” should instead read “This study was supported by the National Natural Science Foundation of China (31100268 to Peng Chen, 31370604 to Bo Zheng);”.

## References

[1] Wang Y, Pang C, Li X, Hu Z, Lv Z, Zheng B, Chen P (2017). Identification of tRNA nucleoside modification genes critical for stress response and development in rice and *Arabidopsis*. BMC Plant Biol.

