# Supplementary material for: Identification of tRNA nucleoside modification genes critical for stress response and development in rice and Arabidopsis
Source: BMC Plant Biol. 2017 Dec 21;17:261. doi: 10.1186/s12870-017-1206-0 (PMC5740945; doi:10.1186/s12870-017-1206-0)
Supplement: Supplementary file 5 — LC-MS/MS chromatogram in MRM mode for authentic nucleosides. The Y axis indicated signal intensity for a particular Q1 and Q3 ions. Numbered peaks are 1: U; 2: C; 3: G; 4: A; 5: D; 6: Ψ; 7:Cm; 8: m5C; 9: ac4C;,10: Am; 11: m1A; 12: m2A; 13: m6A; 14: m6t6A; 15: Gm; 16:m7G; 17: m1G; 18: m2G; 19:m2 2G; 20: Um; 21: m5U; 22: ncm5U; 23: I; 24: m1I; 25: t6A. Figure S2. Eukaryotic tRNA and methylated nucleosides. (A) tRNA cloverleaf structure with methylated nucleosides shaded in gray. The abbreviated name of modified nucleosides was shown in Abbreviations section. (B) Chemical structure of methylated nucleosides and their corresponding MTase enzymes in S. cerevisiae. Figure S3. Multi sequence alignment of methyl-pyrimidine MTases in group I. The conserved amino acids were marked with blue boxes. A high conserved site Gly(G) was highlighted in red (PPTX 399 kb) [file 12870_2017_1206_MOESM5_ESM.pptx]

## Slide 1
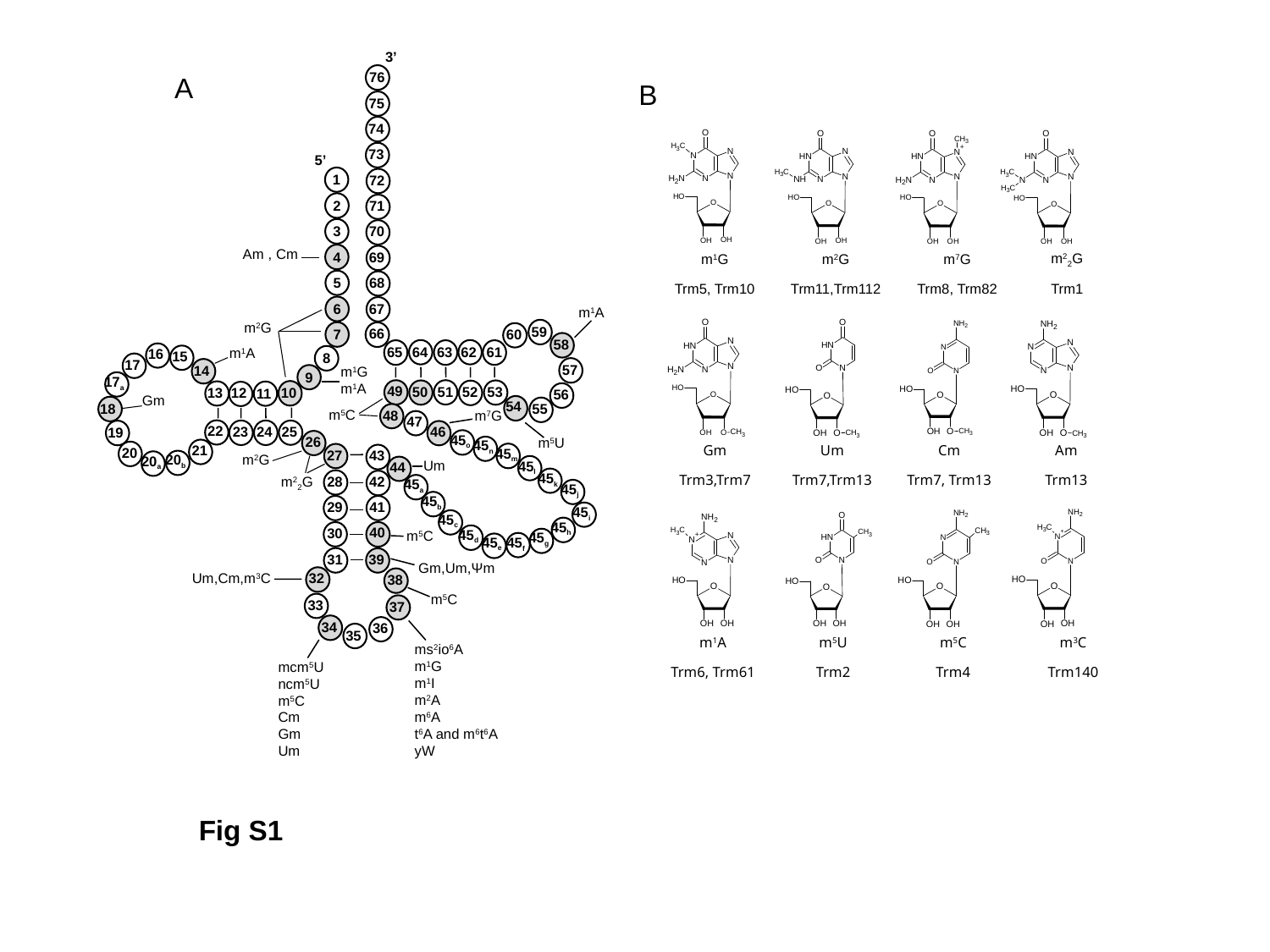

3’
76
75
74
73
5’
72
1
71
2
70
3
Am , Cm
69
4
68
5
67
6
m1A
m2G
59
66
60
7
58
62
64
63
65
61
m1A
16
15
8
17
57
14
m1G
m1A
9
17a
49
51
50
52
53
13
12
10
11
56
Gm
54
18
55
48
m5C
47
m7G
22
24
25
46
23
19
45o
26
45n
m5U
21
20
45m
27
43
m2G
20b
20a
45l
44
Um
45k
28
42
m22G
45a
45j
45b
41
29
45i
45c
45h
40
30
45d
45g
m5C
45e
45f
39
31
Gm,Um,Ψm
32
38
Um,Cm,m3C
33
m5C
37
34
36
35
ms2io6A
m1G
m1I
m2A
m6A
t6A and m6t6A
yW
mcm5U
ncm5U
m5C
Cm
Gm
Um
A
B
| m1G | m2G | m7G | m22G |
| --- | --- | --- | --- |
| Trm5, Trm10 | Trm11,Trm112 | Trm8, Trm82 | Trm1 |
| Gm | Um | Cm | Am |
| --- | --- | --- | --- |
| Trm3,Trm7 | Trm7,Trm13 | Trm7, Trm13 | Trm13 |
| m1A | m5U | m5C | m3C |
| --- | --- | --- | --- |
| Trm6, Trm61 | Trm2 | Trm4 | Trm140 |
Fig S1

## Slide 2
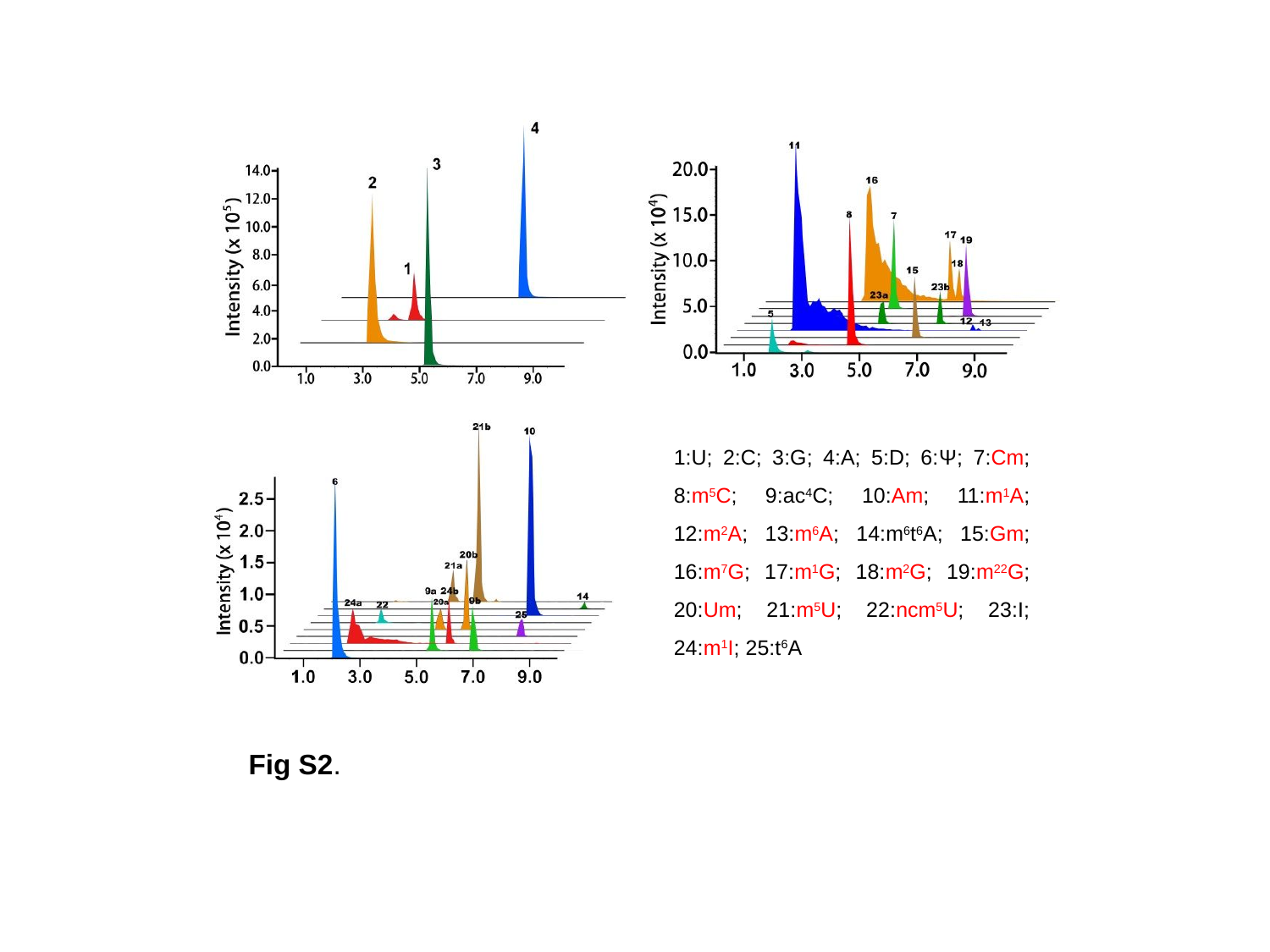

1:U; 2:C; 3:G; 4:A; 5:D; 6:Ψ; 7:Cm; 8:m5C; 9:ac4C; 10:Am; 11:m1A; 12:m2A; 13:m6A; 14:m6t6A; 15:Gm; 16:m7G; 17:m1G; 18:m2G; 19:m22G; 20:Um; 21:m5U; 22:ncm5U; 23:I; 24:m1I; 25:t6A
Fig S2.

## Slide 3
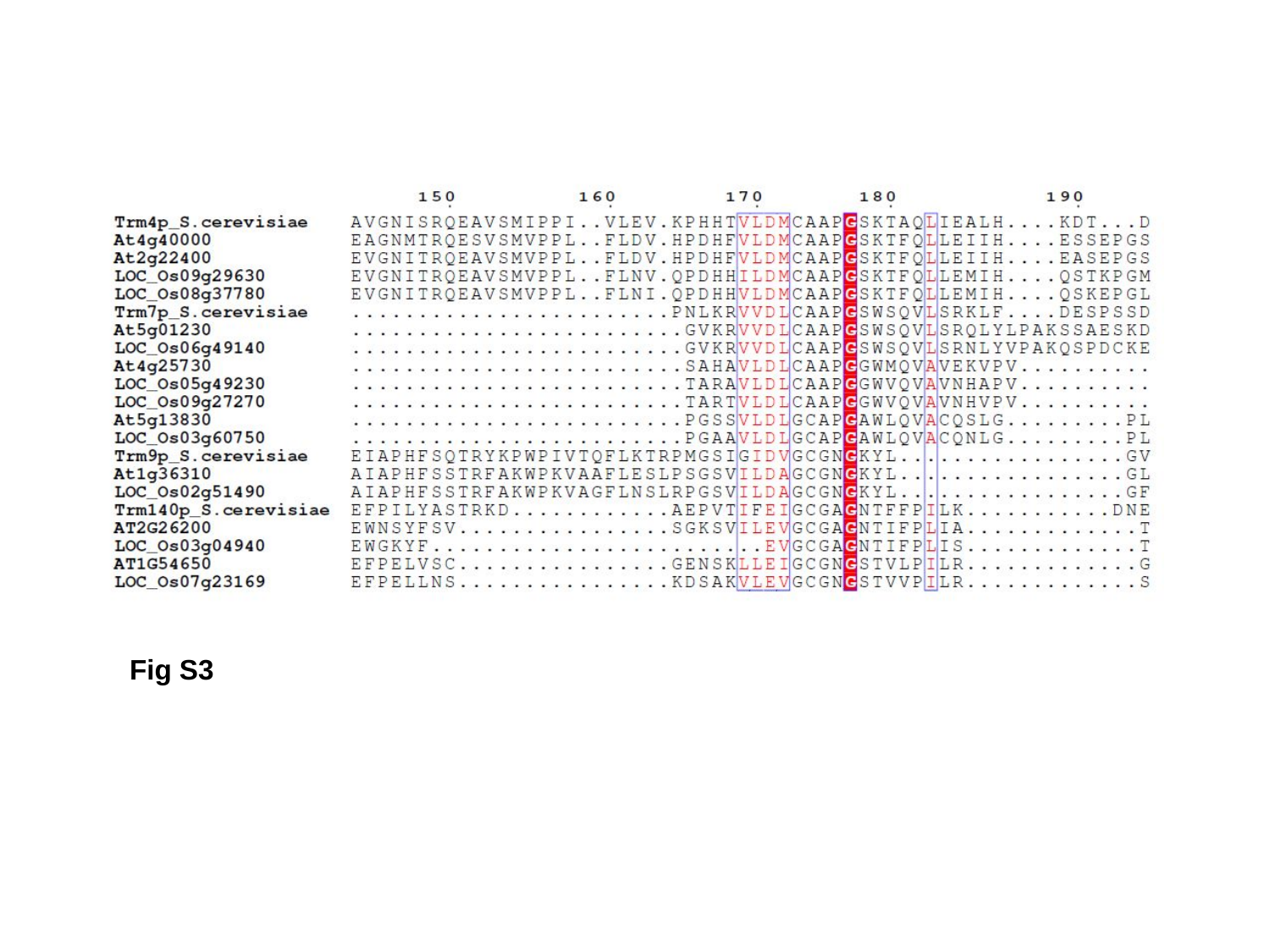

Fig S3
